# The non-catalytic binding of auxin, cytokinin and salicylic acid by tomato Phi class glutathione transferases: insights from computational modelling

**DOI:** 10.1007/s11103-026-01681-2

**Published:** 2026-02-03

**Authors:** Ádám Barnabás Hajnal, Ágnes Gallé, Jolán Csiszár

**Affiliations:** 1 Department of Plant Biology, Faculty of Science and Informatics, University of Szeged, Közép fasor 52, Szeged, 6726 Hungary; 2 Doctoral School of Biology, Faculty of Science and Informatics, University of Szeged, Szeged, Hungary

**Keywords:** GST ligand binding sites, Plant hormones, Protein–ligand interactions, AI protein modelling, Molecular docking, MD simulations

## Abstract

Glutathione transferases (GSTs, EC 2.5.1.18) are a diverse enzyme family involved in various cellular processes, regulated by several stimuli, including plant hormones (phytohormones). Additionally, GSTs can also influence hormone levels, as certain GSTs, particularly members of the Phi class GSTs (GSTFs) have been reported to directly bind small phytohormones (auxin, cytokinin, and salicylic acid). However, the structural aspects of these interactions remained unresolved. It has been documented that an *Arabidopsis thaliana* GSTF, AtGSTF2, possesses unique non-catalytic ligand-binding sites (L sites). This study focuses on the orthologous tomato (*Solanum lycopersicum*) GSTFs (SlGSTFs) and explores the connection between this non-catalytic ligand-binding function and phytohormones. Using modern *in silico* techniques, such as protein modelling by AlphaFold, molecular docking, and molecular dynamics (MD) simulations, we provide the first insights into the possible phytohormone-binding ability of SlGSTFs. In particular, SlGSTF5 shows significant potential for a novel phytohormone binding function, thereby broadening the potential roles of plant GSTs. Our work also includes a simple protocol for modelling and analysing protein-ligand interactions, offering valuable insights into the role of individual plant GSTs.

**Supplementary Information:**

The online version contains supplementary material available at 10.1007/s11103-026-01681-2.

## Key message

This study predicts tomato GSTs’ direct phytohormone binding, precisely SlGSTF5 with auxin, and presents a simple protocol using protein modeling and molecular dynamics simulations.

**Supplementary Information:**

The online version contains supplementary material available at 10.1007/s11103-026-01681-2.

## Introduction

Glutathione transferases (GSTs) are a heterogeneous superfamily of proteins involved in various intracellular processes. Their most characteristic feature is their glutathione-dependent activity, as these enzymes facilitate the nucleophilic attack of reduced glutathione (GSH) on the electrophilic sites of numerous molecules. GSTs are typically highly upregulated in response to both biotic and abiotic stresses, aligning with cellular functions in primary and secondary metabolism, stress signalling and tolerance, and detoxification of xenobiotics as phase II enzymes (Csiszár et al. 2014; Horváth et al. 2019).

In plants, GSTs can be classified into 14 classes based on their gene and protein sequences, among which the tau (GSTU), and lambda (GSTL) classes are plant-specific (Labrou et al. 2015). The Phi class GSTs (GSTF) were also considered to be plant specific as well for decades, however some GSTFs were identified in fungi, myxobacteria, and protists too (Munyampundu et al. 2016).

GSTFs are homodimer enzymes, known for their important protective role, for example, in the stress response of plants due to their significant glutathione-dependent peroxidase activity, but it was suspected that they might also possess unique non-catalytic ligand-binding ability (“ligandin” role), which has been documented on animal GST classes (Alpha & Mu classes) in the 1970s (Levi and Arias 1969; Ketley et al. 1975; Sylvestre-Gonon et al. 2019). In plants, Ahmad et al. (2017) directly described this ligandin function of GSTFs, using X-ray crystallography. In that study, the structural basis for the binding of several small-sized ligands has been revealed, which led to the identification of two new characteristic non-catalytic ligand-binding sites on the *Arabidopsis thaliana* (L.) AtGSTF2 homodimer protein, the so-called L sites (L1 site and L2 site).

Another interesting aspect is that some studies have previously reported that certain plant GSTs can bind phytohormones with small size and a simple aromatic ring structure. Zettl et al. (1994) identified the auxin-binding ability of the aforementioned AtGSTF2, and Bilang and Sturm (1995) reported that a GST isolated from *Hyoscyamus muticus* (L.) was able to bind both synthetic and natural forms of auxin. Moreover, Gonneau et al. (1998) described the cytokinin binding of a GST from *Nicotiana plumbaginifolia* (Viv.). It was later discovered that these GSTs belong to the Phi class of GSTs. Another direct phytohormone binding is known among GSTFs: Tian et al. (2012) described the binding of salicylic acid by several *Arabidopsis* GSTFs (AtGSTF2,8,10,11). Although these biochemically/*in vitro* defined phytohormone bindings are valuable findings, unfortunately, no structural information regarding the exact mechanism of these bindings was available for any of the cases.

Tomato (*Solanum lycopersicum* L.) is one of the most widely cultivated vegetable crops in the world, with an annual yield of more than 186 million tons (FAOSTAT: https://fao.org/statistics/en/), and it is also an important model plant in plant biology. In tomato, 89 GST sequences (SlGSTs) have been identified, among them five genes are belonging to the Phi class: *SlGSTF1* (Solyc02g081340.2), *SlGSTF2* (Solyc06g009020.2), *SlGSTF3* (Solyc06g009040.2), *SlGSTF4* (Solyc09g074850.2), *SlGSTF5* (Solyc12g094430.1) (Csiszár et al. 2014; Islam et al. 2017).

Tomato GSTF (SlGSTFs) enzymes have been featured in several studies, in which various changes at their gene expression levels are known, suggesting their protective role against various stress effects and other processes (Csiszár et al. 2014; Islam et al. 2017; Outchkourov et al. 2018; Gallé et al. 2021; Zhu et al. 2024). Although a significant amount information about SlGSTFs (especially at transcriptional level) is now available in the literature, there is a gap in knowledge regarding the detailed functional mechanisms of these enzymes.

Nowadays, there are numerous *in silico* techniques available for modelling proteins and the interactions between proteins and other molecules. A significant advancement in this area of technology is AlphaFold, which has been widely recognized for its impact on structural biology (Nobel Prize in 2024, Demis Hassabis and John Jumper). This technology allows for the prediction of the spatial structure of proteins with high accuracy solely from amino acid sequences (Jumper et al. 2021). Moreover, as GSTs within the same class exhibit significant conservation in amino acid sequences and structure (Dixon et al. 2002), so the highly precise prediction of the 3D structures of GSTs becomes more straightforward if there are experimentally determined structures of other orthologous GSTs.

Studying the molecular interactions of proteins with various molecular docking techniques have also become widespread, enabling the prediction of the three-dimensional binding of one or more molecules. During molecular docking, multiple different binding poses can be modelled, which are then ranked according to different parameters (“scoring”) in pursuit of the most descriptive and realistic mode of interaction (Agarwal and Mehrotra 2016). However, the nature of proteins and molecular interactions is fundamentally not static as they can take many different spatial conformations with different energy states, which are influenced by a variety of physicochemical parameters. To understand these processes, the dynamic structural changes of biomacromolecules can be studied using molecular dynamics (MD) simulations. This computational technique enables sampling the dynamic “evolution” of a system at atomic resolution in extremely small timesteps (femtoseconds), which is essential for understanding, e.g., the stability of protein–ligand complexes (Shukla and Tripathi 2020).

The root mean square deviation (RMSD) and root mean square fluctuation (RMSF) are the most important numeric parameters when evaluating MD simulations. RMSD allows the calculation of the spatial movement of selected molecules (e.g., the heavy atoms of a protein–ligand complex) compared to their initial position and conformation under the simulations. The stability of the studied system can be generally specified from the average and standard deviation of the obtained RMSD values; in which, the lower the value, the more stable the studied system is. RMSF shows residue-wise fluctuation the selected parts of a protein in the MD trajectory. Similarly to RMSD, the lower RMSF values represent greater stability in the investigated system (Shukla and Tripathi 2020). Thus, RMSD and RMSF analysis are relatively simple but extremely informative methods to gain information about the spatial and temporal stability of a protein–ligand complex over time.

In this research, we aimed to perform an *in silico* study of tomato GSTFs, with a particular focus on the interactions between selected phytohormones with small size, simple aromatic ring structure (auxin, cytokinin, and salicylic acid) and GSTF enzymes. Using AlphaFold models, targeted molecular docking, and MD simulations, we successfully adopted a method where, through a simple RMSD, RMSF and protein–ligand interaction analysis, it can be determined which GST isoenzymes and small ligands are expected to have time-stable interactions. This approach provides the most detailed phytohormone-binding properties of tomato GSTs to date, specifying their potentially novel functions.

## Materials and methods

### Generating the SlGSTF protein homodimer models

According to Csiszár et al. (2014), the amino acid sequences of five GSTFs in tomato were collected from the Sol Genomics Network database (https://solgenomics.net/). The sequences of the previously described phytohormone-binding GSTs were collected from the UniProt database (https://www.uniprot.org/). The used amino acid sequences can be found in Online Resource 1 Table. The homodimer models of SlGSTF proteins were generated utilizing AlphaFold (Jumper et al. 2021). The protein modelling was conducted on the ColabFold (v1.5.5) platform (Mirdita et al. 2022), following the brilliant method described by del Alamo et al. (2022), thereby effectively sampling the structural fluctuations of the whole GST enzymes whilst eliminating structural inaccuracies in the models. This application of AlphaFold was also described by Nussinov et al. (2023) as “reverse pharmacophore” modelling, which presents AlphaFold as an effective tool for ligand-centred mapping of protein binding sites. The L site binding-pockets were identified in UCSF Chimera (Pettersen et al. 2004) from the structural alignment of the SlGSTF AlphaFold homodimer models with the crystal structure of AtGSTF2 with different ligands (PDB IDs: 5A4U, 5A5K, 5A4V, 5A4W). To identify acceptable L site structures, the generated models were filtered based on RMSD values calculated from the structural alignment of residues forming the L site binding pockets in the protein pairs. The used reference structure was the known structure of AtGSTF2 (PDB ID: 5A4U) in complex with indole-3-aldehyde (chosen as the reference ligand due to its structural similarity to active form of phytohormones). The alignment was performed using the PyMOL Molecular Graphics System (version 2.5.8, Schrödinger, LLC), and the models with the lowest RMSD were selected for the molecular docking experiments (separate models for L1 and L2 site variants). The structural evaluation of these models was performed in MolProbity (Williams et al. 2018) (Online Resource 1 Table). To identify residues responsible for specific ligand binding, *in silico* mutant proteins for the selected SlGSTFs were generated via SWISS-MODEL (Schwede et al. 2003) using the appropriate AlphaFold models as templates.

### Multiple sequence and structure alignment of GSTFs

The multiple sequence and structure alignment analysis of the selected GSTFs was carried out using UCSF Chimera, with the corresponding 3D structures aligned using the MatchMaker and Multialign Viewer tools. The protein structures used in the analysis were as follows: the generated SlGSTF models, the known crystal structure of AtGSTF2, and the predicted structures of the previously mentioned hormone-binding GSTFs from the AlphaFold Database (models IDs, amino acid sequences and structural evaluations can be found in Online Resource 1 Table).

### Protein–ligand docking

Several naturally occurring plant hormones were selected for this work. The chosen plant hormones are some of the most notable natural auxin forms: indole-3-acetic acid (IAA), indole-3-pyruvic acid (IPA), indole-3-butyric acid (IBA), 2-naphthylacetic acid (NAA) (Bajguz and Piotrowska 2009); the two cytokinin forms with the highest occurrence in tomato: N6-(2-isopentenyl)adenine (IP), trans-zeatin (TZ) (Keshishian et al. 2018); and salicylic acid (SA). We selected benzoic acid (BA) and tryptophan (TRP) as negative control ligands. BA was selected because of it is known for negatively affecting auxin signalling (Zhang et al. 2018), moreover, it shows great structural similarity to SA. TRP was selected for its similarity to the structure (especially in the aromatic rings) of auxin and cytokinin hormones. Ligands were prepared for docking using the AcePrep tool at PlayMolecule.com (Torrens-Fontanals et al. 2024), while proteins were prepared with UCSF Chimera’s Dock Prep tool. Molecular docking was performed using the default command in GNINA (v1.3) (McNutt et al. 2021), defined by the ligand binding modes of the homologous L sites in the reference structure (PDB ID: 5A4U). A positive control docking experiment was also carried out with IAA on the experimentally determined structure of AtGSTF2 (Ahmad et al. 2017), due to the highly similar structure of the IAA auxin and the bound indole-3-aldehyde ligand (5A4U). The ligand poses for further investigation were selected by the absolute binding affinity value predictions of Kdeep (Jiménez et al. 2018) and GNINA (McNutt et al. 2021), in accordance with the reference docking results.

### Molecular dynamics simulation, trajectory, RMSD, RMSF and protein–ligand interaction analysis

MD simulations were used to test all protein–ligand interactions following the protocol of Kato et al. (2021), with minor modifications, moreover, we applied extended MD simulations as well. These authors demonstrated that short (5–10 ns) MD simulations can also be effective for the selection of relevant protein–ligand interactions using RMSD calculations. They illustrated this approach by assessing various molecular docking binding modes with various proteins, showing that this method can help in filtering out irrelevant results—demonstrated on one GST protein as well (Kato et al. 2021). (Characteristically high RMSD values were observed for inappropriate binding modes.) We selected this method, since given that the sizes of the L site binding-pockets are significantly smaller than the substrate-binding sites of GSTs, hence the potential variability of binding modes during molecular docking is also considerably limited. Our protocol was implemented using the OpenMM engine and AMBER force fields (Arantes et al. 2021). Prior to the MD simulations, the protein–ligand complexes underwent a 20,000-step structural optimization (energy minimization/equilibration step), followed by a 10 ns long production phase for all protein–ligand models to identify stable interactions by RMSD. Additional 90 ns (100 ns total) long production phase was applied for the selected protein–ligand models. In these MD simulations, the time step was set to 2 fs, and the simulation temperature was maintained at 300 K, at constant 1 bar pressure (NPT ensemble). For classical molecular force field calculations, the AMBER ff14SB (protein) and AMBER GAFF2 (ligand) force fields were applied. The solvent filled the space around the protein surface within an 8 Å radius inside a cubic box, containing TIP3P water molecules and a 0.1 M salt concentration with neutralizing sodium and chloride ions. The RMSD plotting of the protein–ligand complexes and clustering was performed with the MD Movie tool in UCSF Chimera. RMSF calculations were performed via MDAnalysis (v2.10.0) (Michaud‐Agrawal et al. 2011). The protein–ligand interactions were investigated using Protein–Ligand Interaction Profiler (PLIP) (Schake et al. 2025) from the average structures represented by the top three largest trajectory clusters (if possible).

### Statistical analysis of the protein–ligand RMSD plots

RMSD was the key selection parameter in our study. Statistical analysis of the RMSD plot datasets (IAA with AtGSTF2 as positive control; BA, TRP on AtGSTF2, also BA, TRP and phytohormones with SlGSTFs) was carried out in SigmaPlot (v12.0, Systat Software Inc., USA) by Holm-Sidak test (One Way ANOVA), and the differences were considered significant if *p* ≤ 0.05. During the comparison of the RMSD plots of phytohormone bindings to the positive control (IAA with AtGSTF2), results that were statistically different from the control dataset were rejected, and only datasets that were statistically not different were used in the investigations. In the case of negative control ligands (BA and TRP with AtGSTF2 and SlGSTFs), only the results that were statistically different from the positive control datasets were retained; and datasets that were not statistically different were rejected.

## Results

### The structure of SlGSTF enzymes partly differs from that described on AtGSTF2, including the L sites

**Fig. 1:**
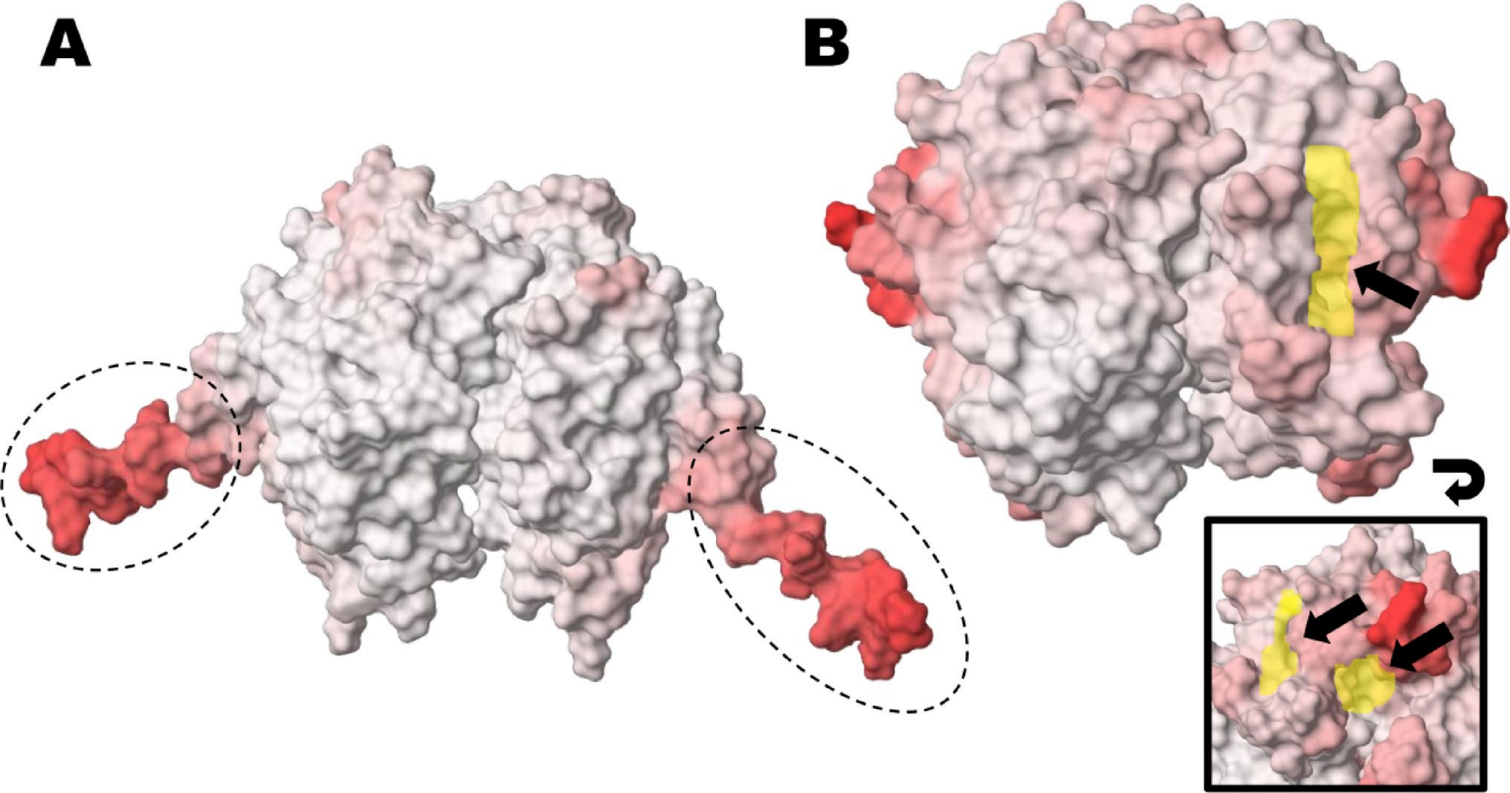
Unique structures among the tomato (*Solanum lycopersicum*) Phi class GSTs (SlGSTFs) AlphaFold models. **a** The model of the SlGSTF1 homodimer, with the C−terminal tails circled by dashed lines. **b** The model of the SlGSTF4 homodimer, with the open surface resulting from the absence of the first segment in the canonical N−terminal domain (marked in yellow, shown from two perspectives). Regions coloured in stronger shades of red represent parts of the protein models that are modelled with higher uncertainty according to AlphaFold. The protein models are shown in PlayMolecule (https://playmolecule.org/)

The structures of SlGSTF models follow the canonical architecture of GSTs, consisting of two identical subunits that form homodimers. Each subunit is composed of the typical N- and C-terminal domains, but with two notable exceptions: SlGSTF1 contains a 14 amino acid-long C-terminal tail (LEMLPGPPKEEVKV) with a currently unknown function and unclear structure (Fig. 1a); and the SlGSTF4 lacks the first segment of the canonical N-terminal domain, including the active site motif, which likely renders its catalytic function and introduces greater uncertainty regarding its overall protein structure (Fig. 1b).

**Fig. 2:**
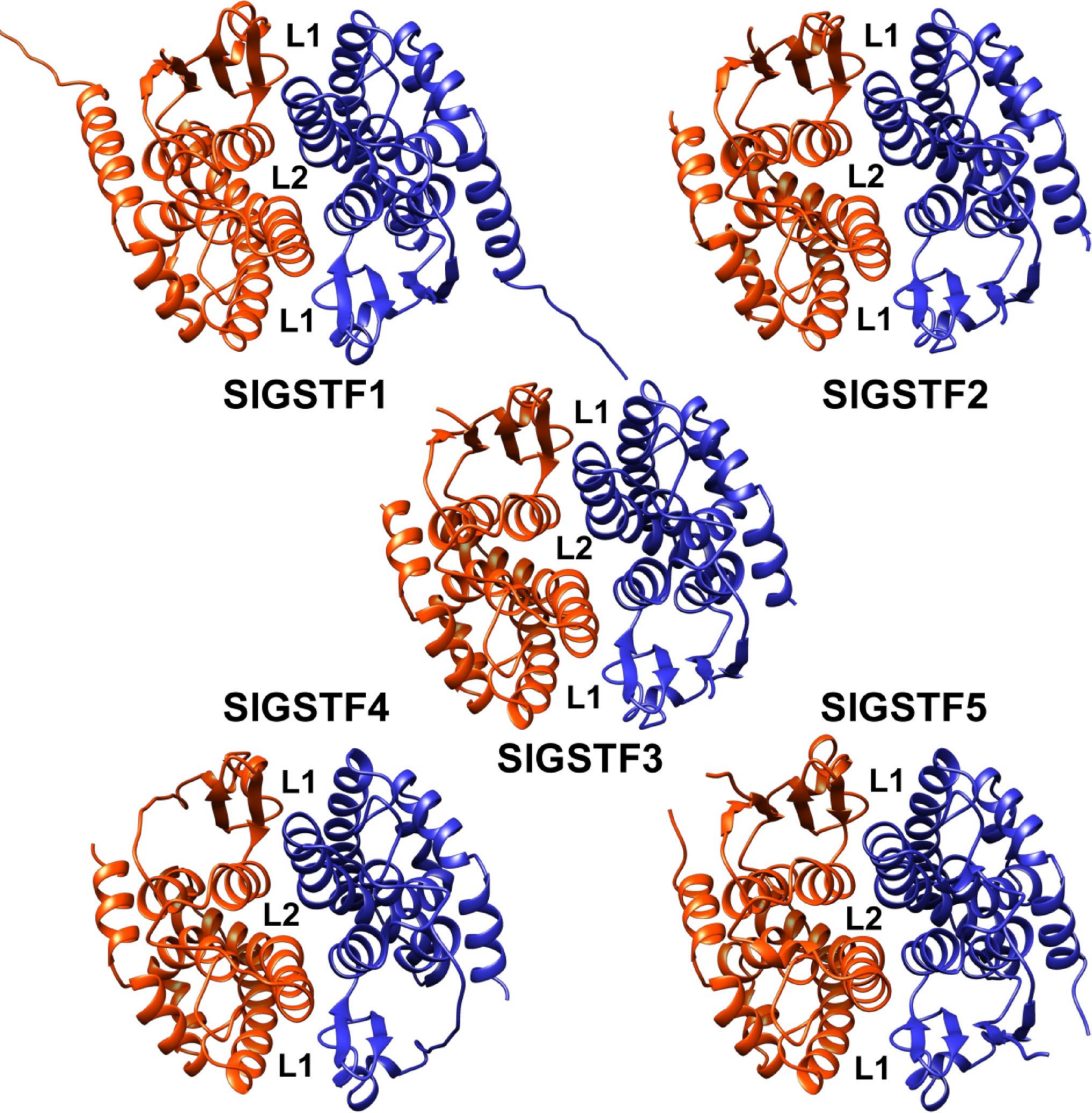
The location of the two non−catalytic ligand binding sites (L1 & L2 sites) on each tomato (*Solanum lycopersicum*) Phi class GSTs (SlGSTFs) AlphaFold models. The two subunits of the SlGSTF homodimers (ribbon diagrams) are coloured in orange and blue. The protein models are shown in UCSF Chimera

We generated a total of 80 distinct conformational models for each SlGSTF dimer using AlphaFold. Among these, we searched for the models with “open” L1 and L2 sites, and one model was selected and used from the conformers for each SlGSTF L site variants in our workflows. (The confidence scores of the selected AlphaFold models can be found in Online Resource 1 Table). The amino acid composition of the SlGSTF L sites compared to the AtGSTF2 is shown in Table 1, and the location of the L sites on each SlGSTF homodimer is shown in Fig. 2.

**Table 1:** The amino acid composition of the SlGSTF L sites, compared to AtGSTF2

AtGSTF2	SlGSTF1	SlGSTF2	SlGSTF3	SlGSTF4	SlGSTF5
*L1 site comparison*					
Pro51	Pro50	Pro50	Pro50	Pro36	Pro52
Phe52	Phe51	Phe51	Phe51	Phe37	Phe53
Lys64	*Arg63*	*Lys63*	*Lys63*	*Lys49*	*Thr65*
Phe66	Phe65	Phe65	Phe65	Phe51	Phe67
Ile99	Leu95	Val95	Ile95	Ile81	Ser98
Ile102	Gln98	Val98	Val98	Val84	Gln101
Gly103	**Trp99**	**Trp99**	**Trp99**	**Trp85**	**Trp102**
Val106	Val102	Val102	Val102	Val88	Ala105
Val150	Ile146	Val146	Val146	Ile133	Val149
Tyr151	–	–	–	–	–
Arg154	Arg150	Arg150	Arg150	Arg137	Arg153
Leu161	–	–	–	–	–
Ala162	–	–	–	–	–
Thr169	–	–	–	–	–
*L2 site comparison*					
Ala70	Ala69	Ala69	Ala69	Ala55	Ser71
Gln73	Arg72	Gln72	Gln72	Gln58	Arg74
Tyr74	Tyr73	Tyr73	Tyr73	Tyr59	Tyr75
His77	Ala76	His76	His76	Gln62	Asp78
Ile94	Leu90	Pro90	Pro90	Pro76	Pro93
Tyr97	Lys93	Met93	Met93	Met79	Lys96
Ala98	Ala94	Ala94	Ala94	Ala80	Ala97
Ala101	Asp97	Ser97	**Tyr97**	Ser83	Asp100

Comparison of the residues forming the general non-catalytic ligand−binding sites (L sites) of the tomato (*Solanum lycopersicum*) Phi class GSTs (SlGSTFs) and the *Arabidopsis thaliana* AtGSTF2 based on their spatial structures. Compared to the AtGSTF2 crystal structure, the residues that significally affected the shape of the L sites on the SlGSTF proteins are marked bold, additional residues which also may be parts of the SlGSTF L1 sites are marked in italics. The crossed-out cells represent residues that do not participate in the formation of the SlGSTF L sites due to spatial block

**Fig. 3:**
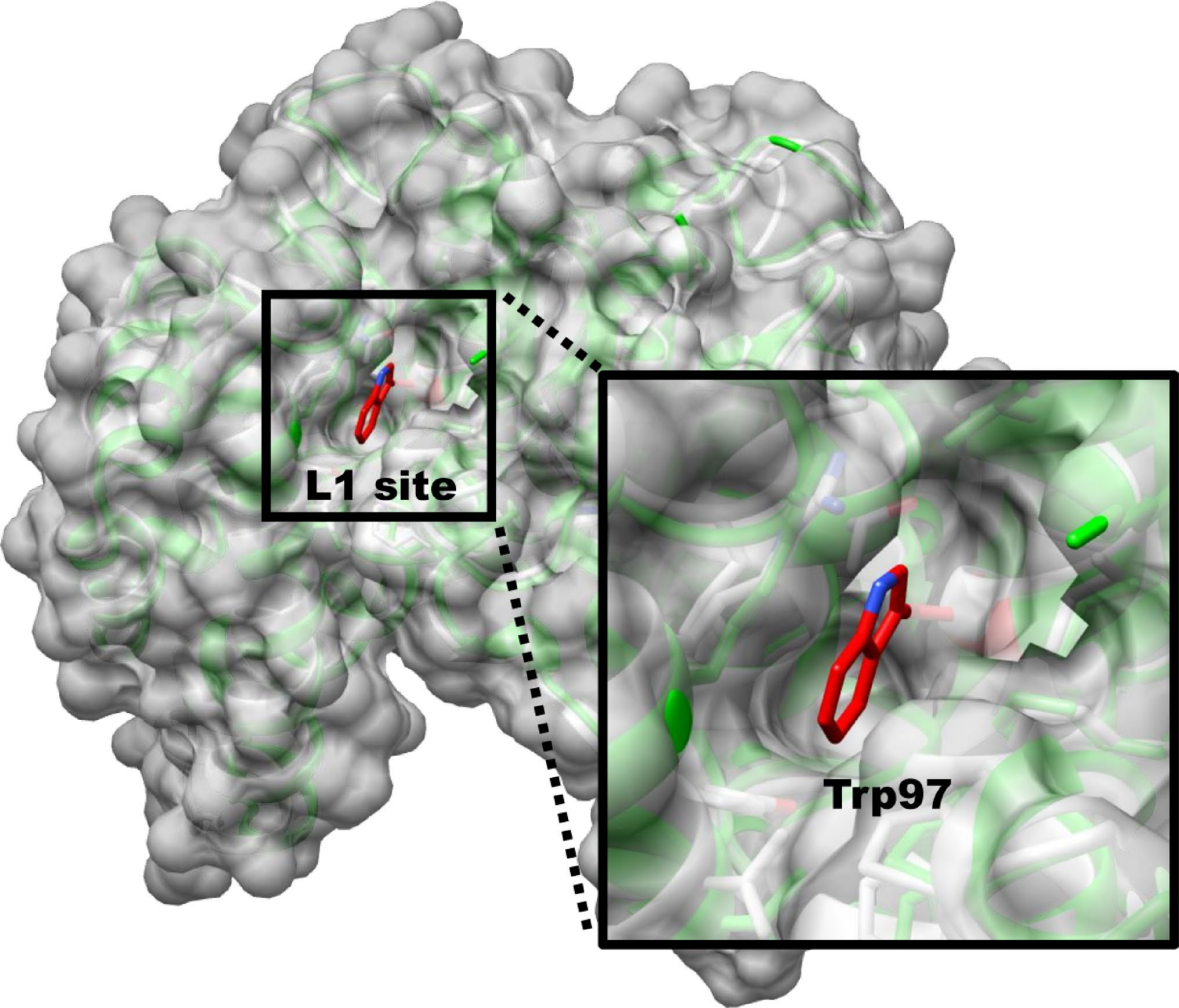
Representation for the effect of one amino acid difference in the structure of the non−catalytic ligand−binding site 1 (L1) on a selected tomato (*Solanum lycopersicum)* Phi class GST (SlGSTF). The 3D model of SlGSTF2 homodimer (coloured green, surface not shown) was aligned with the *Arabidopsis thaliana AtGSTF2* crystal structure (coloured white, surface shown), highlighting the impact of the Trp97 residue on shape of the binding pocket (coloured in red). The protein models are shown in UCSF Chimera

Based on structural comparisons, the L2 site structures identified in the SlGSTF2,3,4 homodimers vary minimally from that found in the AtGSTF2 structure (similar amino acid composition), but the composition of SlGSTF1 and SlGSTF5 L2 sites is more diverse. The structure of all SlGSTF L1 sites differ significantly from AtGSTF2 in one specific location. This difference is that the Gly amino acid at position 103 in AtGSTF2 is analogous to Trp in in all five SlGSTFs, a residue with a large side chain (Table 1, residue codes in bold text). This difference drastically changes the shape of the L1 site, resulting in the SlGSTF enzymes having “shallower” binding pockets. As a consequence, interactions with certain residues in the ligand-binding pocket of SlGSTFs are not possible in due to the spatial occupation of Trp, compared to AtGSTF2 (example shown in Fig. 3). However, considering the rest of the residues in our models, the L1 sites seemed similar to the reference structure.

One additional residue was considered part of these special SlGSTF L1 sites, as their spatial position may enable interactions with certain ligands (Table 1, residue codes underlined). No similar structural difference was detected at the SlGSTF L2 sites, except the case of SlGSTF3, where the bulky side group of Tyr97 (analogous to Ala101 in AtGSTF2) physically blocked the entire binding pocket on the L2 site at each conformer variant (Table 1, residue code in bold text). Therefore, in the case of SlGSTF3, we were only able to examine the L1 site in this work.

### The L sites are potentially present among all the selected plant GSTFs

**Fig. 4:**
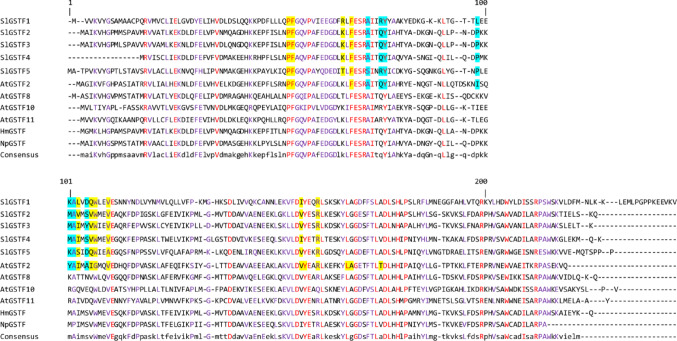
Multiple sequence alignment (MSA) of tomato (*Solanum lycopersicum*) Phi class glutathione transferases (SlGSTF1, SlGSTF2, SlGSTF3, SlGSTF4, SlGSTF5) and GSTF amino acid sequences with known hormone−binding abilities (*Arabidopsis thaliana*: AtGSTF2, AtGSTF8, AtGSTF10, AtGSTF11; *Hyoscyamus muticus*: HmGSTF; *Nicotiana plumbaginifolia*: NpGSTF). Highly conserved residues (80% or greater) are displayed in purple and capitalized, and the completely conserved residues shown in red. The identified residues involved in the formation of the L1 site are highlighted in yellow, and in the case of the L2 site in blue. The sequence alignment was formatted in Microsoft Office 365 Word

MSA analysis showed that 32.24% of the investigated sequences are highly conserved (80% or greater). Also most importantly, the MSA revealed that the sequence segments containing the identified L site residues are among the most conserved regions within the selected GST sequences. The result of the MSA can be found in Fig. 4, and the identified residues involved in the formation of the L site binding pockets on the SlGSTF and AtGSTF2 sequences are also shown (L1 site residues are highlighted in yellow, L2 in blue).

### The template-based protein–ligand dockings followed by short and extended MD simulations revealed potential hormone-binding ability among SlGSTFs

Molecular docking and short MD simulations were performed on the AtGSTF2 dimer with IAA in the two L site binding pockets to use them as positive controls for evaluating the analyses conducted on SlGSTF enzymes. The molecular docking revealed that IAA occurred in the L1 & L2 site binding pockets like the small ligands in the aforementioned structures of AtGSTF2, and this was also supported by the short MD simulations.

**Fig. 5:**
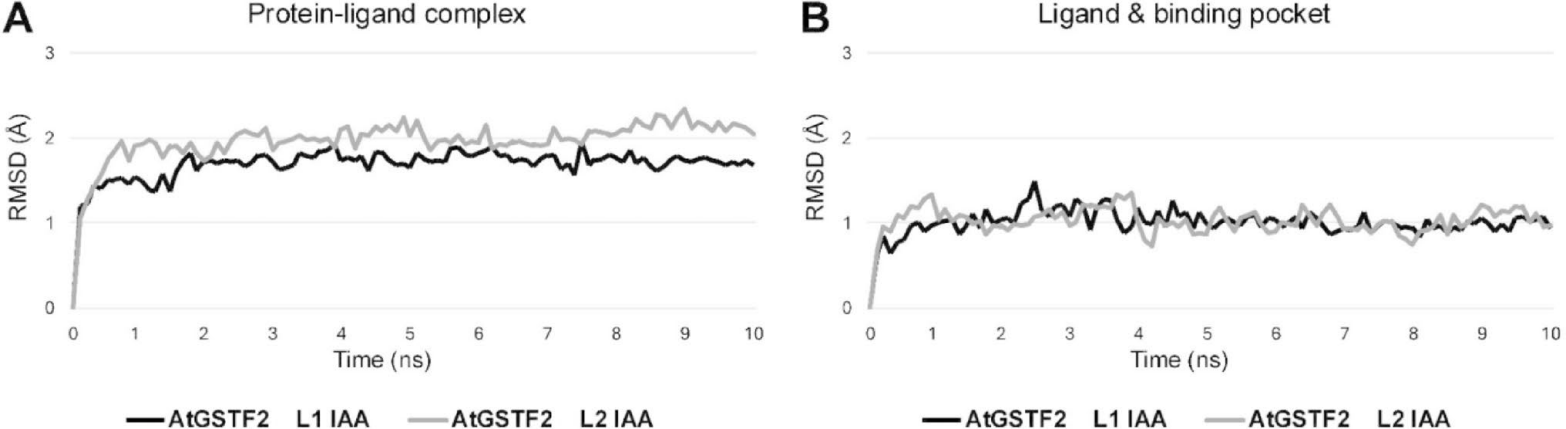
The calculated root mean square deviation (RMSD) plots of **a** the whole *Arabidopsis thaliana* AtGSTF2 dimer and indole−3−acetic acid (IAA) complex, and **b** IAA and the residues within a 5 Å zone from the ligand (the binding pocket) during 10 ns MD simulations. The RMSD plots display the spatial movement of the selected atoms during the molecular dynamics simulations, reflecting the stability of the studied system. The graphs were created in Microsoft Office 365 Excel

The RMSD plots of AtGSTF2 with IAA protein–ligand complexes can be visualized as logarithmic curves with low deviation, whose linear sections can be described by a line of low slope, indicating great temporal stability of the system (Online Resource 2 Table). The average RMSD (Å) values, calculated including heavy atoms, were 1.699 ± 0.136 for the L1 site simulation and 1.983 ± 0.185 for the L2 site simulation (Fig. 5a). However, in our experience, the temporal stability of the investigated complex can be more accurately defined if the calculation of the RMSD plots is limited only to the heavy atoms of the ligand and the binding pocket (the residues within a 5 Å zone from the ligand), thus allowing a precise and exclusive tracking of the changes in the ligand's position and the spatial movement of the residues within ligand-binding pocket (Fig. 5b; Online Resource 2 Table). The average RMSD values in the zones defined by the IAA ligand and the binding pocket were 1.012 ± 0.129 (L1 site), and 1.031 ± 0.132 (L2 site). RMSD for the all protein–ligand complexes were calculated in the same way in the subsequent analyses as well.

In our work, we performed a total of 98 MD simulations. In the first phase of the workflow (designed to screen for potential protein–ligand interactions), we conducted 65 short (10 ns) MD simulations involving SlGSTFs and phytohormones. Protein–ligand systems, that did not statistically deviate from the positive control, were selected and carried forward for further analysis. In most cases, we observed weak and unstable interactions between the SlGSTFs and the selected phytohormone ligands (usually accompanied by high and/or increasing RMSD values), where the different ligands were found in various positions within the L sites during the short MD simulations, and often dissociated from the protein after a short time period (data not shown). However, we found statistically supported, temporally stable protein–ligand interactions in 9 cases, compared to the positive control MD simulations (IAA and AtGSTF2). (Table 2).

**Table 2:** Stable protein–ligand interactions of phytohormones (auxin, cytokinin, salicylic acid) with the L sites of SlGSTFs identified in the initial screening phase using 10 ns MD simulations

Protein ID	Site	Bound phytohormone and type	Ligand and binding pocket RMSD (Å) (+ SD)
SlGSTF1	L2	Indole-3-acetic acid (IAA), auxin	1.113 ± 0.103
SlGSTF1	L2	Trans-Zeatin (TZ), cytokinin	1.092 ± 0.120
SlGSTF2	L1	Trans-Zeatin (TZ), cytokinin	1.028 ± 0.136
SlGSTF2	L2	Indole-3-acetic acid (IAA), auxin	0.991 ± 0.157
SlGSTF3	L1	2-Naphthylacetic acid (NAA), auxin	1.064 ± 0.161
SlGSTF4	L1	Trans-Zeatin (TZ), cytokinin	1.061 ± 0.257
SlGSTF4	L1	Salicylic acid (SA)	1.093 ± 0.170
SlGSTF5	L2	Indole-3-butyric acid (IBA), auxin	0.956 ± 0.128
SlGSTF5	L2	2-Naphthylacetic acid (NAA), auxin	0.971 ± 0.189

Stable protein–ligand interactions between tomato (*Solanum lycopersicum*) Phi class glutathione transferases (SlGSTFs) and their statistically proven phytohormone ligands (auxin, cytokinin, salicylic acid) within their non−catalytic ligand−binding sites (L sites), identified during the initial screening phase using 10 ns MD simulations. The root mean square deviation (RMSD) averages were calculated including the heavy atoms of the ligands and the residues within a 5 Å radius of the ligand

In the second phase of our study, the 9 previously selected SlGSTF and 2 AtGSTF2 protein–ligand systems were subjected to extended 100 ns MD simulations to further assess their stability under the same parameters as before. During these prolonged simulations, 6 tomato protein-hormone interactions—in addition to the AtGSTF2 positive controls (AtGSTF2 L1, L2 with IAA)—remained non-dissociated: SlGSTF1 L2 site with TZ, SlGSTF2 L1 site with TZ, SlGSTF2 L2 site with IAA, SlGSTF3 L1 site with NAA, SlGSTF4 L1 site with TZ, and SlGSTF5 L2 site with IBA. At this stage, we used negative control ligands (BA and TRP in molecular docking and 10 ns long MD simulations) on the 6 SlGSTF and the AtGSTF2 L sites in order to filter false positive interactions.

Based on the results of the long (100 ns) MD simulations, RMSD values generally increased compared to the shorter runs (Online Resource 2 Table). Therefore, we also calculated the RMSF values of the amino acids forming the binding pockets to more effectively identify the most stable protein–ligand interactions. For ligands localized at the L1 site, the lowest (most stable) RMSF values were observed for SlGSTF2 L1 with TZ, while for ligands at the L2 site, the most stable interaction was SlGSTF5 L2 with IBA (Online Resource 3).

Regarding the results obtained with negative control ligands, both the statistically supported RMSD values and the visual outcomes of the simulations showed the same result. The BA and TRP ligands dissociated from both the SlGSTF2 L1 site and the SlGSTF5 L2 site within a few nanoseconds (drastically increasing RMSD values—see in: Online Resource 2 Table), therefore the SlGSTF2 L1 site and the SlGSTF5 L2 site were selective towards phytohormones. An important observation was that, for the reference AtGSTF2 L1 and L2 binding sites, no significant difference in RMSD values was found between the binding of IAA and the negative control ligands BA and TRP. However, these negative control ligands dissociated from the L1 site during extended 100 ns MD simulations, while they remained stably bound at the L2 site (RMSD plots in Online Resource 2 Table). Notably, the L1 site with IAA produced RMSD values similar to those observed for SlGSTF2 and SlGSTF5, suggesting that IAA binding to the L1 site may exhibit specificity.

By comparing the RMSF values from the long MD simulations and the RMSD values from simulations with negative control ligands (where RMSF values were not accurately interpretable for protein–ligand interactions due to ligand dissociation), we selected the SlGSTF2 L1 TZ and SlGSTF5 L2 IBA protein–ligand interactions for further analysis in the subsequent workflows.

In the third phase of the study, PLIP was used to investigate the key interactions in the protein–ligand structures derived from MD trajectory clusters. We identified residues that formed secondary chemical bonds stronger than hydrophobic interactions with the ligands in the selected clusters (Table 3). For AtGSTF2, Arg154 was found as the most important residue in the binding of IAA at the L1 site (salt bridge and π-cation interaction). In the case of SlGSTF2, among the amino acids forming the L1 binding pocket, Lys63 established one hydrogen bond with the TZ ligand, and we also identified Ser149 as another residue interacting through hydrogen bonding (one bond). (The orthologous residue, Ala153 in AtGSTF2, likely does not contribute to ligand binding due to its physical distance.) The output files (.prmtop and.dcd files) from the simulations performed with IBA binding to the SlGSTF5 L2 site, and IAA binding to the AtGSTF2 L1 site, as well as the average structure model (as a.pse file) of the IBA binding on the SlGSTF5 L2 site, are available in Online Resource 4.

**Table 3:** Identified interactions between selected phytohormones on the L sites of GSTFs

Residue ID	Interaction type	Contact atom/group of ligand
*AtGSTF2 L1 site with indole-3-acetic acid (IAA), auxin*		
Ile102	Hydrophobic interaction	Aromatic ring C5
Arg154	π-cation interaction	Aromatic rings
Arg154	Salt bridge	Carboxyl group
*SlGSTF2 L1 site with trans-Zeatin (TZ), cytokinin*		
Lys63	Hydrogen bond	Aromatic ring N3H or N9H
Val95	Hydrophobic interaction	Methyl group
Ser149	Hydrogen bond	Hydroxyl group
*SlGSTF5 L2 site with indole-3-butyric acid (IBA), auxin*		
Arg74	Hydrophobic interaction	Aromatic ring C7
Tyr89	Hydrophobic interaction	Butyric group C10
Tyr89	Hydrogen bond	Aromatic ring N1H
Lys96	Salt bridge	Carboxyl group
Ala97	Hydrophobic interaction	Aromatic ring C7

Protein–ligand interactions between *Arabidopsis thaliana* and the selected tomato (*Solanum lycopersicum*) Phi class glutathione transferases (AtGSTF2, SlGSTF2 and SlGSTF5) and their phytohormone ligands (auxin and cytokinin) within their non−catalytic ligand−binding sites (L sites). The protein–ligand interactions were identified via Protein–Ligand Interaction Profiler (PLIP) from the average structures taken from trajectory clustering of 100 ns MD simulations

In the case of SlGSTF5, we also identified two residues with significant interaction capabilities regarding the binding of IBA. One is Lys96, which forms one salt bridge, and the other is Tyr89, a residue contributing to the binding pocket that interacts through one hydrogen bond (Table 3). This residue is unique due to its spatial positioning and polarity, significantly contributing to the formation of the ligand binding-pocket. In orthologous GSTFs, this position is predominantly occupied by apolar leucine or isoleucine, or, for example, in SlGSTF1, by Thr86, which is also a polar amino acid, but a much smaller residue in terms of spatial occupancy.

Residues with significant interactions in the available clusters during the binding of BA and TRP were also examined. Regarding the amino acids listed earlier, only partial overlap was observed in the interaction capabilities of SlGSTF2 Lys63 and SlGSTF5 Lys96 with the control ligands (data not shown). Based on the results obtained from screening protein–ligand interactions identified using PLIP, *in silico* mutant protein variants of SlGSTF2 and SlGSTF5 were generated using SWISS-MODEL, substituting the aforementioned residues with alanines in these AlphaFold models (SlGSTF2 K63A S149A and SlGSTF5 Y89A K96A).

Molecular docking and 10 ns MD simulations on these mutant variants were performed, utilizing the two selected hormones and negative control ligands to examine the changes in RMSD, RMSF values, and the composition of protein–ligand interactions. In the case of the SlGSTF2 K63A S149A variant, the TZ binding persisted after amino acid substitution, and dissociation of negative control ligands ceased. However, RMSD averages for TZ and TRP binding were significantly higher compared to the positive control (TZ: 1.318 ± 0.224, TRP: 1.251 ± 0.216 (Å); Online Resource 2 Table). While the overall RMSF average of the binding pocket remained relatively unchanged, the effects of residue substitutions were evident in elevated RMSF values and averages at Ala63 and Ala149 (Ala63: from 0.696 to 1.312, Ala149: from 0.704 to 1.278 (Å); Online Resource 3 Table). PLIP analysis revealed that the modified residues did not participate in ligand binding. For SlGSTF5 Y89A K96A, RMSD increased significantly only for negative control ligands, but their dissociations were missing (BA: 1.460 ± 0.358, TRP: 2.018 ± 0.322 (Å); Online Resource 2 Table). RMSF calculations showed no change in the average values for the entire binding pockets, however the Tyr89Ala substitution resulted in an increased RMSF average for these simulations, while the Lys96Ala substitution at position 96 either did not alter or reduced RMSF (Ala89A/B: from 0.588/0.507 to 0.688/1.040, Ala96A/B: from 0.671/0.991 to 0.974/0.602 (Å); Online Resource 3 Table).

**Fig. 6:**
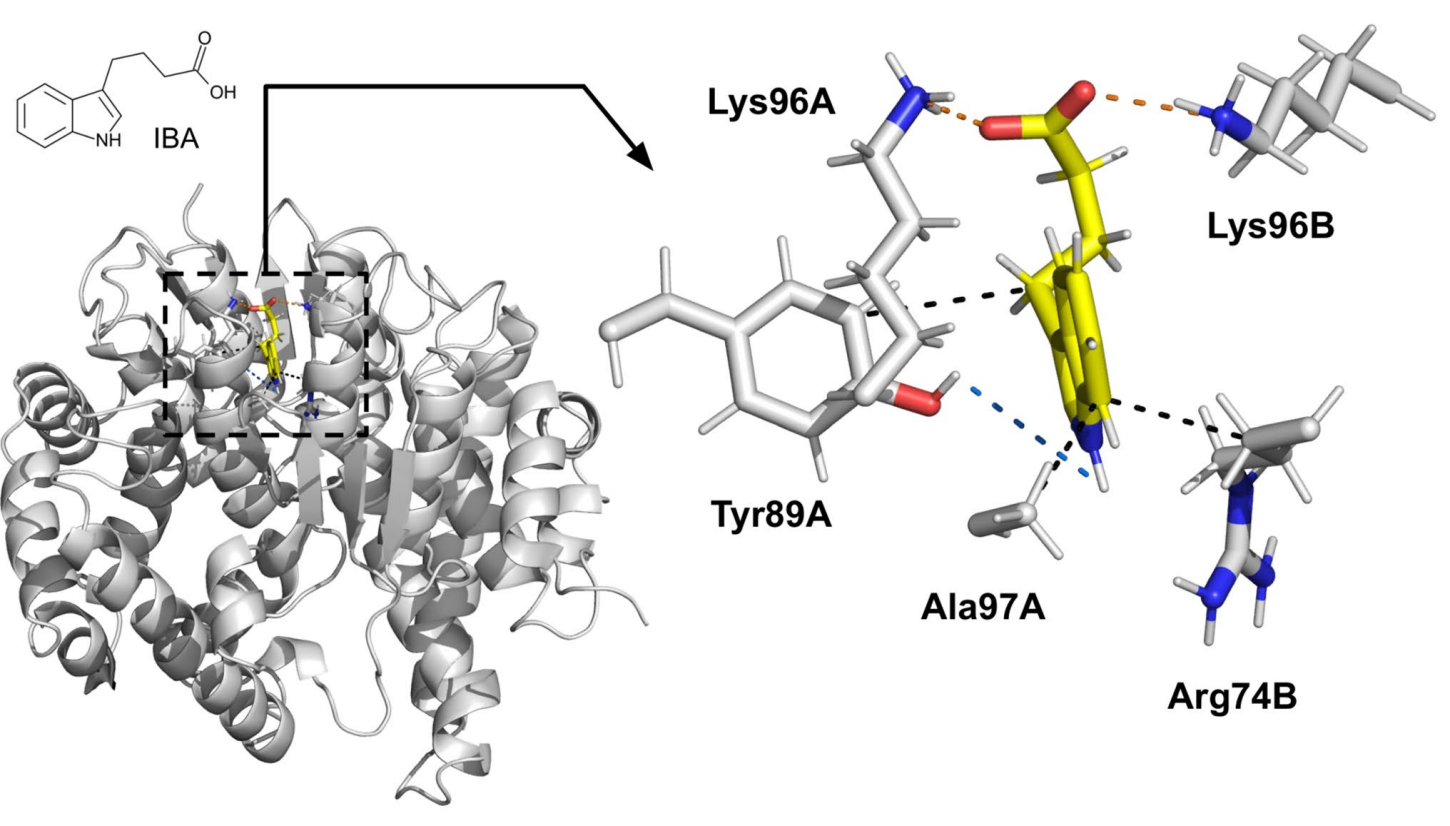
The identified interactions between indole−3−butyric acid (IBA) auxin phytohormone and the residues within non−catalytic ligand−binding site 1 (L1 site) of the tomato (*Solanum lycopersicum*) Phi class glutathione transferase SlGSTF5. The protein–ligand interactions were identified via Protein–Ligand Interaction Profiler (PLIP) from the average structures taken from trajectory clustering of the 100 ns MD simulation. The shown interactions are the following: salt bridge (orange dashed line), hydrogen bond (blue dashed line), hydrophobic interaction (black dashed line). Visualized in PyMOL

In summary, IBA binding to the SlGSTF5 L2 site appears to be the most specific and well-founded interaction in this study (Fig. 6). Stable binding was consistently observed in both 10 ns and 100 ns MD simulations, as evidenced by RMSD values. (In particular, the IBA ligand itself exhibited a remarkably low RMSD of 0.583 ± 0.090 Å.) Additionally, the average RMSF values of the residues forming the binding pocket and the results of simulations performed on *in silico* mutant proteins further support this conclusion. While substitution of amino acids abolished the dissociation of negative control ligands in both variants, only the original IBA ligand binding to SlGSTF5 showed no significant difference in RMSD values compared to the positive control simulation of AtGSTF2 with IAA (considering the ligand and residues within 5 Å), decisively confirming the specificity of the native binding site. This observation is particularly significant given that our findings suggest AtGSTF2 L1 or L2 sites may accommodate a broader range of ligands (supported by the multiple crystallographically determined ligand bindings and our simulation results with negative control ligands).

## Discussion

### The connection between plant GSTs and phytohormones

Phytohormones are biologically active substances that regulate plant growth, development, and stress responses, as well as the activity and function of GSTs (Jain et al. 2010). Certain plant GSTs are known to be induced by phytohormones, including auxins, cytokinins, and salicylic acid, which play key roles in their regulation (Seckin Dinler et al. 2023). Additionally, plant GSTs have been reported to bind these hormones (Zettl et al. 1994; Bilang and Sturm 1995; Gonneau et al. 1998), indicating the possibility of a crosstalk between hormones and enzymes. Notably, some human GSTs have been proven to participate in the biosynthesis of various hormones (leukotrienes and steroids) (Oakley 2011). However, some sources (such as Kumar and Trivedi 2018) assumed the participation of plant GSTs in these processes as well. Currently, no concrete information can be found on this in the literature so far, so the possible role of plant GSTs in the phytohormone biosynthesis is still not clear. Overall, current literature suggests that the relationship between plant GSTs and phytohormones like auxin, cytokinin, and salicylic acid involves the regulation of *GST* gene expression and some direct protein-hormone interactions, although the underlying mechanisms have remained unknown so far.

### AlphaFold2 protein modelling combined with molecular docking offers an easily applicable method to investigate protein–ligand interactions of GSTs

It is undeniable that AlphaFold3 has surpassed its predecessor, AlphaFold2, by demonstrating the ability to model not only protein structures but also complexes involving diverse molecules, such as ligands, nucleic acids, and ions (Abramson et al. 2024). However, combining AlphaFold2 with molecular docking may remain a more practical approach for certain applications. Our workflow is significantly faster, more accessible, and less resource-intensive, since AlphaFold3 currently entails higher hardware demands and restrictive licensing. Additionally, our hybrid method may enable the sampling of biologically relevant weaker interactions as well, whereas AlphaFold3 may overlook metastable ligand poses in favour of a single high-confidence conformation. Altogether, our described protocol offers an easy-to-use and time-efficient method for examining protein-phytohormone interactions in GSTs.

### The simulations with AtGSTF2 raised the potential role of the L1 site in the interactions with indole-3-acetic acid

Kato et al. (2021) successfully demonstrated that short MD simulations (5–10 ns) can be sufficient for selecting favourable ligand poses. In our study, we regarded IAA binding to the AtGSTF2 L1 and L2 sites as the positive control, based on RMSD plots calculated from 10 ns MD simulations (stabilizing logarithmic curves with low deviation). However, results from extended 100 ns simulations and negative control ligand tests indicate that this phytohormone ligand binding cannot be considered fully specific, as we observed the lower RMSD and RMSF averages for other orthologous proteins and negative control ligands (and the existence of other crystallographically identified bound ligands). However, it is important to note that BA and TRP ligands dissociated from the L1 site during the 100 ns MD simulations (data not shown), therefore the binding of IAA at the L1 site of AtGSTF2 is a considerably valuable result. While the involvement of plant GSTs in the regulation of phytohormones was already suspected (Micic et al. 2024), and the structure and binding of certain ligands (including indole-3-aldehyde, structurally very similar to auxins like IAA) on the L sites of AtGSTF2 have been described (Ahmad et al. 2017), this work provides the first explanation for the mechanism behind the binding of the IAA phytohormone to AtGSTF2, which was first reported by Zettl et al. (1994). Considering the L1 site specific for the binding of IAA, numerous chemical bonds and contacts were observed after analysing the average structures from the trajectories with PLIP (Table 3), investigating the potential interactions between AtGSTF2 and other phytohormones might be beneficial. However experimental validation is still required to further describe the exact nature of these interactions. Additionally, this work could serve as reference for further investigations into other unexplained processes associated with plant GSTs. Interestingly, our results may also explain the auxin-binding ability the orthologous *Hyoscyamus muticus* GSTF too (Bilang and Sturm, 1995). This is supported by sequence- and structural similarity of the L sites in these GSTFs (shown in Fig. 4). For example, the RMSD between the heavy atoms of the superpositioned subunits of the AtGSTF2 crystal structure and the HmGSTF model (AlphaFold Database ID: AF-P46423) is 0.555 Å (calculated using UCSF Chimera's MatchMaker tool), that may indicate potential functional analogy, similarly to the study by Georgakis et al. (2020) in the structural comparison of two monocot GSTFs.

### The relationship between tomato GSTFs and certain phytohormones is much stronger than previously thought

While the direct hormone-binding properties of certain GSTFs are already known, there is relatively limited amount of information in the literature on the direct interaction between tomato Phi class GSTs and auxin, cytokinin, and salicylic acid, although a few studies provide valuable insights into the relationship between these components.

Auxin is one of the main phytohormones regulating plant growth and development, and it is also closely linked to plants' stress responses and redox regulation, including GSTs (Hernández-Carranza et al. 2023). Auxin is known to effectively enhance GST activity as safener, and numerous auxin-regulated elements (AuxRR-core) are found in the promoters of many GST genes, solely among SlGSTFs, in the promoter of *SlGSTF5* as well (Csiszár et al. 2014; Islam et al. 2017). In an essential study, Pinto et al. (2023) presented the direct relationship between SlGSTFs and auxins in tomato, studying the effect of the synthetic auxin herbicide 2,4-dichlorophenoxyacetic acid (2,4-D), with special regard to GSTF enzymes. After the treatments, the expression of *SlGSTF4* and *SlGSTF5* genes showed a significant increase in the plant leaves, with a smaller but still significant rise in the roots as well. Based on our results, SlGSTF5's auxin-specific ligand-binding ability (Fig. 6), as well as the aforementioned promoter element and the gene expression induction by 2,4-D, suggests a valid auxin-binding ability, likely originating from the unique structure and amino acid composition of the L2 site on this enzyme (Table 1).

Cytokinins are involved in various processes, from seed germination to plant senescence, with TZ standing out among the different cytokinin forms due to its high bioactivity and widespread, universal effects among plants (Bajguz and Piotrowska 2009). While cytokinins are closely related to stress (Keshishian et al. 2018) and the increased levels of stress-induced reactive oxygen and nitrogen species (Tognetti et al. 2017), very little is known about the relationship between GSTFs and cytokinins, except for the direct cytokinin-binding discovered by Gonneau et al. (1998). Interestingly, Keshishian et al. (2018) found no induced or repressed tomato GSTFs among the cytokinin-regulated genes in their comprehensive study, only in the stress-related genes. Based on the limited information and available literature, it can be assumed that although the TZ-binding ability observed in the case of SlGSTF2 is worth considering, but this might be a minor role for these enzymes.

Salicylic acid is another highly important phytohormone in plant stress defence and various plant processes, certainly influencing GSTs as well (Csiszár et al. 2014; Horváth et al. 2023). In the context of SA and tomato GSTFs, Gallé et al. (2021) explored the effects of low-concentration SA on the expression of numerous GST genes, highlighting that the *SlGSTF1* and *SlGSTF4* genes exhibited a dramatic increase in gene expression following a 24-h 0.1 mM SA treatment in two tomato varieties. The effect of higher SA concentrations (1 mM) on the expression of certain *SlGSTF* genes is also known, such as the marked induction of *SlGSTF2* expression, which was accompanied by increased GST activity too (Czékus et al. 2023). Notably, Islam et al. (2017) identified salicylic acid response elements in the promoters of *SlGSTF1*, *SlGSTF3*, and *SlGSTF4* genes. Furthermore, *SlGSTF4* is closely related to *AtGSTF8* (Csiszár et al. 2014), which is an early SA-inducible stress marker GST and one of the SA-binding GSTs (Tian et al. 2012). Although we did not identify stable, long-lasting binding of SA to the SlGSTF proteins in our study, the literature and results from short MD simulations may suggest that further investigation of certain SA conjugates (George Thompson et al. 2017), particularly in the context of their potential role in transport processes may be warranted.

Based on our results and the available literature, SlGSTF5 seems to be the most remarkable phytohormone-binding members among tomato GSTFs. The importance of this enzyme can be further emphasized by data from the Protein Abundance Database (PaxDB: https://pax-db.org/), which shows that SlGSTF5 is in the top 10% of proteins present in tomato leaves (data by McWhite et al. 2020). It is possible that the elevated presence of SlGSTF5 could be associated with a potential transport function on the L sites in the case of indole-3-butyric acid. However, this hypothesis requires experimental validation.

## Conclusions

The description of AtGSTF2 structure with the discovery of the GSTF L sites have provided significant insights into the non-catalytic ligand-binding function of plant GSTs, expanding the potential roles of GSTFs. Our approach, using AlphaFold2 combined with deep learning-enhanced molecular docking, effectively predicted protein structures and ligand interactions, although it inherently lacks native ligand binding prediction, unlike the newer version, AlphaFold3. This study attempted to predict the direct binding of phytohormones to SlGSTFs, but based on our *in silico* simulations, only the binding of indole-3-butyric acid by SlGSTF5 seems plausible. While our results for SlGSTF5 enzyme are notable, further experimental validation is also required to describe the exact nature of these interactions. Nonetheless, this study firstly reports the predicted direct phytohormone-binding of a tomato GST, SlGSTF5, and the explanation of AtGSTF2's IAA binding on the L1 site. We also successfully developed a protocol combining protein modelling, molecular docking and MD simulations, and through simple methods like RMSD, RMSF and protein–ligand interacion analysis, we have provided valuable information on the role of individual GSTs with previously unclear functions.

## Supplementary Information

Below is the link to the electronic supplementary material.

Supplementary Material 1

Supplementary Material 2

Supplementary Material 3

Supplementary Material 4

## Data Availability

The models and datasets generated for the current study are not publicly available due to because this would require revealing the personal private contact information and data deposits, but they are available from the corresponding author on reasonable request.
